# Supplementary material for: An electrodiffusive, ion conserving Pinsky-Rinzel model with homeostatic mechanisms
Source: PLoS Comput Biol. 2020 Apr 29;16(4):e1007661. doi: 10.1371/journal.pcbi.1007661 (PMC7213750; doi:10.1371/journal.pcbi.1007661)
Supplement: Attachment — Submitted filename: Response_letter.pdf [file pcbi.1007661.s001.pdf]

Dear Editor,

We hereby resubmit our manuscript "An electrodiffusive, ion conserving Pinsky-Rinzel model with homeostatic mechanisms" by Marte J. Sætra, Gaute T. Einevoll, and Geir Halnes for publication in PLoS Computational Biology.

We thank the editor and reviewers for their time and effort and are grateful for their useful comments and suggestions put forward to improve the manuscript. We have followed most of them, as described below, and we think that the manuscript is substantially improved as a consequence.

Below follows a detailed response to the points raised by the reviewers, using the color coding:

Blue: Reviewer´s comments.

Black: Author´s response.

Red: Citations of added/edited text in the revised version of the manuscript.

Reviewer #1: The paper applies the Kirchoff-Nernst-Planck framework (a method for modeling electrodiffusion) to a reduced CA3 cell model. The original two compartment model is augmented with two additional extracellular compartments, modeling of both the intracellular and extracellular potentials, electrodiffusion of K+, Na+, Cl- and Ca2+ and several homoeostatic mechanisms. The model qualitatively matches the original, but unlike the simple model they started from the electrodiffusive model can be driven into depolarization block by excessive stimulation or depolarize when the homoeostatic mechanisms are impaired. This novel and innovative approach will be useful for the studying the mechanisms underlying pathological conditions like ischemia and epilepsy, that can lead to spreading depolarization.

Comment: We thank the reviewer for this positive evaluation of our manuscript.

Reviewer #1: The sodium calcium exchanger takes in 3 sodium for every calcium ion at the cost of 1 ATP. Why do you have it exchanging 2 sodium ions instead? Eq 67 & 71.

Comment 1: We wanted to maintain the intracellular Ca2+ decay similar to that of the original PR model (eq. 58), since this was important for the membrane potential dynamics (via Ca2+ activated K+ channels). In the original PR model, the decay was electroneutral, i.e., Ca2+ decayed without (directly) affecting the membrane potential. Since we in our (edPR) model derived the membrane potential from ion concentrations and their associated charge, a Ca2+ decay (in isolation) would cause a reduced charge density and thus a drop in the membrane potential. To avoid that, we made the decay electroneutral through the phenomenological model where 1 Ca2+ was exchanged with 2 Na+. If we instead used 3 Na+ for each Ca2+, the decay would again amount to a net membrane current, which would affect the voltage dynamics in a way which would be inconsistent with the original PR model. The electroneutral decay in our

model could putatively be obtained in a real system not through a single mechanism (such as the 3Na+/1Ca2+ exchanger), but rather the joint effect of several mechanisms.

Action: We added the following clarification of this in the Methods section (lines 672-674):

"Putatively, this phenomenological model for the decay could represent the joint effect of several mechanisms in a real system, such as the Cai/3Na exchanger, a Caleakage current, SERCA pumps, etc."

Comment 2: PS. We tried to replace the electroneutral exchange with a 3Na+/1Ca2+ exchanger, but found that it made the model unstable. This could probably be mended by introducing additional mechanisms to counteract this instability, but we did not try this.

Reviewer #1: The tortuosity is mentioned in the methods section, the value results from an increase in path length as ions diffusion around obstacles. Does this make sense for your model? If so, why haven't you also included the free volume fraction?

Comment 1: Thank you for this insightful comment. We have been thinking quite a lot on these matters ourselves, and believe that we have made the best choice in this regard. Firstly, the free volume fraction is indeed included explicitly in the model. That is, the extracellular space has half the volume of the intracellular neuronal space, which is in agreement with the typical neurons/extracellular/glial volume fractions of 0.4/0.2/0.4 in neural tissue. Hence, we do account for the fact that intracellular and extracellular dynamics occur in reduced fractions (0.4/0.2) of the total tissue volume. We did not state this clearly in the original manuscript, but have done so in the revised version:

Action: In the Methods section, introducing Table 1, we now state (lines 563-565):

"Since typical neuronal/extracellular/glial volume fractions in neuronal tissue are 0.4/0.2/0.4 \cite{Chen2000}, we let the extracellular space be half as voluminous as the intracellular neuronal space."

Comment 2: Regarding the tortuosity, it makes sense to include it as long as we do not consider the geometry of the system to be explicit (down to a microscale level). That is, coarse-grained transport processes over the distances considered in our model, i.e., from soma layers to dendritic layers, do not occur through free (unhindered) extra- or intracellular space, but will face the obstacles represented by the tortuosity.

Reviewer #1: This seems like it could be an issue for interpreting the models boundary conditions as equivalent to period boundary conditions for a synchronized population, as the population would have a relatively large extracellular space.

Comment: We hope that our response to your previous comment convinced you that this is not an issue for the interpretation of the boundary conditions. To comment further on this, the model geometry is so that it uses the tissue averaged quantities "intracellular volume per neuron" and "extracellular volume per neuron". When we

model just a single neuron, the soundness of the assumption that the extracellular boundaries are closed depends on the (assumed) activity of the surrounding population. If the surrounding population is completely inactive, the assumption is poor, as extracellular ions would then diffuse and drift extracellularly away from or towards the one active neuron. If all neighbors are completely synchronized with the one (modelled) neuron, the assumption is perfect: Since the extracellular concentrations/potentials would be the same everywhere in a plane, there would be no transport in the lateral direction, and our model would then represent the entire population. That is, the model is scalable so that we could multiply all volumes and areas by the same constant factor and obtain the same dynamics.

Reviewer #1: Figure 4: Are the ion concentrations as stable as the somatic membrane potential?

Do all the simulations remain stable over the whole +/-15% parameter change or are some slowly depolarizing, like a less extreme 8A? (or hyperpolarizing).

Comment: We admit that the sensitivity analysis included in the original manuscript was somewhat incomplete. What hindered us from making a more complete analysis was that the variables varied on two different time scales. That is, when we change the model parameters, what happens is that the membrane potential first reaches a quasi-steady state (not too far from the original resting potential) rather rapidly. After this, the quasi-steady-state potential and the concentrations change on a much slower time scale, approaching a final steady state. Unfortunately, the sensitivity analysis requires many simulations, and due to memory issues we were not able to run it for longer than the 15 s shown in the original manuscript, which only allowed us to explore the existence of a "fairly stable" quasi-steady-state. For the +/- 15 % parameter changes we considered, it typically took the system more than 100 s to reach the final steady state, something that we observed in selected test simulations. Hence, we could not do a satisfactory analysis of the final steady state by using that approach.

Action: Your comment inspired us to replace our original analysis with a simpler (and more standard) sensitivity analysis, where we increased/decreased single parameters one by one by +/- 15 % and plotted the resulting dynamics of the membrane potential and all ion concentrations (The new Figure 4). Although these (new) simulations do not cover combinatorial cases, i.e., cases where several parameters are varied at the same time, they indicate clearly that the model is not highly sensitive to parameter choices, which was the main point we wanted to make. That is, for modest +/- 15% parameter perturbations it settles at a new steady state in the proximity of the default one. In the new Figure 4 we show that both the membrane potential and ion concentrations approach a new steady state.

Reviewer #1: Figure 6 and 7: Plotting changes freely diffusion calcium on the same scale as sodium is not useful. Why not plot the states as you did for figure 8?

Comment: Thank you for pointing this out. We agree.

Action: In the revised version, we have changed the relevant figures (3,6,7,8 & 9) so that a given panel now shows the concentration of a single ion species in all four

compartments. (In the original version, a given panel showed all four ion concentrations in a single compartment).

Reviewer #1: Figure 8: Why do you turn off all of the homoeostatic mechanisms, rather than just those two that depend on ATP?

Comment: We agree that it would be more natural to turn off only the ATP-dependent mechanisms. When we did this, the response was qualitatively similar to what we found with all homeostatic mechanisms turned off, but the dramatic events (bursts and depolarization block) occurred later.

Action: In the revised version, we have replaced Figure 8 with an updated version which shows the dynamics in the system when only the ATP-dependent homeostatic mechanisms are turned off. The old simulations were discarded.

Reviewer #1: Why does the intracellular dendritic calcium differ so much from the intracellular somatic calcium?

Comment: This has two reasons: Firstly, in the edPR model, as in the original PR model, only the dendritic membrane contained Ca2+ channels and Ca2+ dependent mechanisms. Secondly, the equilibration of intracellular Ca2+ between the soma and dendrite was slow because only 1% of the intracellular was free/unbuffered.

Action: In the revised version we have tried to explain both these points carefully:

In line 173-175 of the Results section, we explained the first point by adding the sentence:

"During AP firing, the ion concentrations varied in a jigsaw-like fashion in all compartments, except for Ca2+, which returned to baseline between each AP and showed notable variation only inside/outside the dendrite since the soma contained no Ca2+ channels. "

In line 354-363 of the Results section, we explained the second point by adding the passage:

"As the Ca2+ dynamics in Fig. 8G may seem counterintuitive, we here give some additional explanation of it. During the burst and initially stages of the depolarization block, the dendritic Ca2+ channels were open. Extracellular Ca2+ then diffused from the soma towards the dendrite where it flowed into the neuron. This resulted in a low Ca2+ concentration in both extracellular compartments and a high Ca2+ concentration in the intracellular dendritic compartment. The reason why the intracellular Ca2+ equilibrated more slowly than the extracellular, was that, by assumption, only 1% of the intracellular Ca2+ concentration was unbuffered and free to diffuse (see Methods), hence, the effective intracellular concentration gradient was a factor 100 lower than it "appears" in Fig. 8E."

Reviewer #1: Initial conditions: How did you adjust the ion concentrations to arrive at stable initial condition? Would the model be unstable if you used initial with concentration from the literature? e.g. Ions in the Brain, Somjen. GG, 2002.

Comment 1: Thank you for commenting on this. We realize that we were unclear about this point, and that our study would be more convincing if we could start it out using initial values taken from the literature.

Action: In the revised version, we have taken the initial ion concentrations from the the book "Neuroscience" by Purves et. al. 2012, re-calibrated the model, and re-run all simulations based on the new initial concentrations. The outcome of the calibration is presented in the new Table 5.

Comment 2: With the new initial values, the simulation outcome and conclusions remained qualitatively similar to those in our original submission. The model was thus not highly sensitive to the choice of initial concentrations. However, the membrane conductances, pump rates and concentrations will together determine the resting membrane potential that the cell will settle on. We tried different sets of initial concentrations taken from the literature, and some choices gave unrealistically high or low resting potentials. Some even lead to "resting potentials" above the firing threshold, so that the model became spontaneously active. Of course, we could in principle always obtain the desired resting potential for any combination of initial concentrations by re-tuning the passive conductances/pump rates. In the revised version, we used the initial concentrations from Purves et al. 2012, because they gave rise to the resting potential that we wanted without us having to re-tune conductances.

Reviewer #1: Also, why didn't you use the post calibration states as your initial conditions? What do the first 15s of the simulations look like?

Comment: We agree that this would be the best way to do it.

Action: In the revised version, all simulations start with the post-calibration states. In the sub-section called "Initial conditions" (in Methods), we carefully explain the choice of initial concentrations and the calibration of the model.

Reviewer #1: Does this mean the initial (post-calibration) conditions for the sensitivity analysis were different for each simulations?

Comment: As we explained in the response to one of your previous comments, we replaced the original sensitivity analysis with a one-at-a-time approach. All runs start with the same initial concentrations (and membrane potential). What the sensitivity analysis then shows is essentially the model calibrating to new the steady states associated with the new parameterizations.

Reviewer #1: Minor issues/typos;

Reviewer #1: Line 392: ICRP model -- only mentioned here.

Comment: This was a typing mistake, and we noticed the same error in line 116 as well.

Action: Both instances replaced with "the edPR model".

Reviewer #1: Figure 10: figure legend is not the same as the caption.

Action: Corrected.

Reviewer #1: Table 1 caption: Two As

Action: Corrected.

Reviewer #1: Table 1: Both intracellular volumes don't correspond to a sphere with radius 7um.

Comment: We had exchanged two parameter values in the table.

Action: The revised Table 1 has the correct values.

Reviewer #2: This paper proposed an electrodiffusive Pinsky-Rinzel (edPR) model, which is "a minimal neuronal model" with two neuronal compartments (a soma and a dendrite), plus two extracellular compartments (outside soma and outside dendrite), by adding homeostatic mechanisms and ion concentration dynamics. The edPR model doesnot only reproduce the membrane potential dynamics of the PR model, but also accounts for changes in neuronal firing properties due to deviations from baseline ion concentrations. They expect the model to be important because it opens for more detailed mechanistic studies of the pathological conditions associated with large changes in ion concentrations such as epilepsy and spreading depression.

Overall, the article is well organized and presented. I have some minor comments here.

Comment: We thank you for these positive remarks on our manuscript.

Reviewer #2: 1. The statement of "the first multicompartmental neuron model that in a biophysically consistent way does account for the effects of ion concentration variations " is too strong. For example, Kager et al 2000 is a morphologically explicit multicompartmental model with ion concentration variations.

Comment: It is true that Kager et al. 2000 (and several other multicompartment models that we cited) do account for some effects of ion concentration variations. However, as we explain in the results subsections titled "Loss in accuracy when neglecting electrodiffusive effects on concentration dynamics" and "Loss in accuracy when neglecting electrodiffusive effects on voltage dynamics" these previous models are not entirely consistent: They include ions that diffuse without affecting electrical potentials, although they should, since moving ions carry electrical currents. Also, they have electrical currents that do not affect ion concentration dynamics, although they should, since electrical currents in the brain are mediated by ions.

Action: In the revised version we have tried to be more specific regarding what we mean by biophysically consistent. We have replaced the "too bold" statement with:

"... the first multicompartmental neuron model that accounts for ion concentration variations in a way that ensures complete and consistent ion concentration and charge conservation."

Reviewer #2: 2. What is the advantage of using the two-compartmental model compared with one-compartment model or morphologically explicit model?

Comment 1: The difference between single compartment models and models with two or more compartments is quite fundamental: it is the difference between not having versus having a spatial resolution. Having no spatial resolution, single compartment models can for example not be used to model the interplay and difference between somatic and dendritic signalling, which was an important topic in the original work by Pinsky and Rinzel. Also, models with no spatial resolution do not produce extracellular (or intracellular) potentials, and modelling these is necessary for understanding experimental recordings of such potentials. Hence, several fundamental properties of neural dynamics can not be addressed with single compartment models.

Action 1: To be clear on this, we added the following passage in the introduction (lines 71-75):

"Single compartment models are useful in many aspects. However, in order to represent morphological features of neurons, such as e.g., differential expression of ion channels in the soma versus dendrites, or account for transport processes in the space inside or outside neurons, one needs models with more than a single compartment."

Comment 2: The difference between having two versus having several compartments is less fundamental. Technically, it would be straightforward to expand the edPR formalism by adding additional compartments if one were to desire a higher spatial resolution.

Action 2: In the discussion, we added the sentence (lines 501-502):

"Technically, it would be straightforward to increase the number of compartments (i.e., the spatial resolution) in the model."

Reviewer #2: 3. The model has two neuronal compartment compartments (a soma and a dendrite), it is a little confused how did the authors investigate the ionic diffusion along the axons in the statement of "investigate the consequences of neglecting the effect of ionic diffusion (along dendrites and axons) on the electrical potential".

Comment: Thank you for spotting this. It was a typing mistake.

Action: We have corrected the sentence to

"... neglecting the effect of ionic diffusion (along dendrites) on the electrical potential"

Reviewer #2: 4. Table 3, T is body temperature at 36C (309.15K).

Action: In the revised version, we have followed your suggestion, and now write:

"...we get an absolute temperature of 309.14 K, corresponding to a body temperature of 36 C."
